# Efficacy of an anonymous, preventive, personalized online counselling to improve shift workers’ sleep quality: a randomized controlled trial

**DOI:** 10.1038/s41598-026-64210-7

**Published:** 2026-07-29

**Authors:** Lukas Retzer, Elmar Graessel, Monika Feil, Robert Lehmann, Mark Stemmler, Kneginja Richter

**Affiliations:** 1 Centre for Health Services Research in Medicine, Department of Psychiatry and Psychotherapy, Uniklinikum Erlangen, Friedrich-Alexander-Universität Erlangen-Nürnberg, Erlangen, Germany; 2 Chair of Work and Organizational Psychology, Friedrich-Alexander-Universität Erlangen-Nürnberg, Erlangen, Germany; 3 Faculty for Social Sciences, University of Applied Sciences Nuremberg Georg-Simon- Ohm, Nuremberg, Germany; 4 Department for Dementia Research and Nursing Science, University for Continuing Education Krems, Krems an der Donau, Austria; 5 Chair of Psychological Assessment, Quantitative Methods and Forensic Psychology, Friedrich-Alexander-Universität Erlangen-Nürnberg, Erlangen, Germany; 6 CuraMed Tagesklinik Nürnberg GmbH, Nuremberg, Germany; 7 University Clinic for Psychiatry and Psychotherapy, Paracelsus Medical University, Nuremberg, Germany

**Keywords:** Shift work, Sleep, Online counselling, Prevention, Depression, Insomnia, Health care, Neuroscience, Psychology, Psychology

## Abstract

Shift work is associated with an increased risk of sleep disorders, yet tailored prevention and treatment programs remain rare and understudied. Cognitive behavioral therapy for insomnia is the first-line treatment for insomnia in the general population. We thus developed an anonymous online counselling service based on cognitive behavioral principles, adapted it to shift workers’ needs, and evaluated it in a sample of German shift workers from various industries. This prospective, randomized, controlled superiority trial compares two parallel groups: an intervention group that received four personalized counselling messages and completed four weekly sleep diaries, and a waiting-list control-group that completed sleep diaries only during the four-week waiting period. In the Intention to Treat sample (*n* = 66), significant improvements were observed in both groups for the primary outcome, sleep efficiency, and for the secondary outcomes, symptoms of insomnia and depression. Daytime sleepiness did not improve in either group. However, improvements were not significantly greater in the treatment than in the control group. We can thus not demonstrate superiority of the counselling intervention over the control condition, where repeated sleep diary completion and study participation may themselves have had therapeutic effects. Limitations regarding time frame and sample size, as well as implications for research and practice are discussed.

*Trial registration:* The study was registered with the German Clinical Trials Register DRKS under the trial ID DRKS00017777 (https://drks.de/search/en/trial/DRKS00017777). Date of registration: 14.01.2020. This study was registered during the early stages of data collection. Of the 27 participants in the final sample, the first four had initiated data collection at the date of registration. No complete data sets were available at that time.

## Introduction

Shift work is associated with a wide range of adverse health and performance outcomes^1^. Although further research on causal pathways is necessary, current evidence suggests it should thus largely be avoided where possible^2^. In practice, however, many industries rely on round-the-clock staffing. Hospitals and other care facilities require continuous personnel to ensure patient care, while sectors such as nuclear energy and offshore drilling rely on constant monitoring to prevent accidents and environmental disasters. Likewise, highly automated industrial production systems continue to depend on human oversight to maintain safety, efficiency, and profitability. As a result, millions of workers face a trade-off between optimal health and occupational demands^3,4^. As with other occupational health hazards, preventive strategies are therefore needed to mitigate negative consequences of shift work^5–7^.

While workers can be protected from noise, chemical fumes, or radiation with adequate safety equipment, protection from the detrimental effects of shift work is less straight forward. Central to these deliberations are the circadian misalignment and resulting sleep disorders, which are often direct consequences of shift work^7^. While medication, bright light exposure, and scheduling optimization have been researched and proven at least partly effective in improving shift worker sleep^7^, the gold standard cognitive behavioral approach to treatment of psychophysiological sleep disorders in the general, non-shift working population^8^ has received limited attention^9,10^. The reasons include high costs, a lack of availability of appropriately qualified therapists, as well as scheduling conflicts^11^. In addition, some contents of the standard cognitive behavioral approach need to be adapted before they can be implemented in this population^12^. Elements such as stimulus control, relaxation techniques, psychoeducation, and cognitive restructuring largely remain applicable. However, the core tool of sleep restriction, i.e. limiting time in bed and adhering to a regular sleep-wake schedule in order to increase homeostatic sleep pressure, create voluntary mild sleep deprivation, and decrease sleep fragmentation, clashes with shift schedules, especially those including rotation. Additionally, some topics, such as light exposure, naps, and caffeine use, likely need to be addressed differently than in the non-shift working population. Finally, the delivery of interventions towards shift workers comes with its own challenges due to the circadian misalignment and resulting scheduling conflicts mentioned above.

Efforts to improve the cost-effectiveness, availability, and flexibility of sleep counselling for the general population have attempted to translate the contents to online settings, with varying, but mostly promising results^13^. Applications of online sleep counselling in shift work exist as well^10^, however, rarely have they (1) employed a randomized controlled trial design, (2) included workers from more than one industry, (3) tailored cognitive behavioral contents specifically to the shift work context, (4) relied on counsellors to personalize and individually guide the intervention instead of algorithms, and (5) facilitated asynchronous and anonymous contact with the counsellors. By addressing these five issues in this study, we attempt to generate a foundation from which valid statements about the effectiveness of personalized, counsellor-guided online sleep counselling in shift work across different industries can be made. While cognitive behavioral therapy for insomnia in the general population is well researched and known to be effective^8^, the same cannot be said for such adapted solutions.

The sixth and final concern we aim to address is the preeminently clinical focus of the literature on online sleep counselling in shift work. While this focus is certainly valid, seeing as 10–23% of shift workers meet the diagnostic criteria for a sleep disorder^1^, we argue that practically all shift workers are at an increased risk of developing a sleep disorder and many report subjective sleep issues and reduced sleep times while not fully meeting clinical criteria^14^. Adopting a more preventive approach to sleep counselling by including these sub-clinical cases may thus help improve the well-being of even more people, especially in the long run. We recognize that this widening of the target population may seem at odds with the lack of qualified therapists we mention above. We argue that severe cases of shift work related sleep disorders need to be temporarily taken off the shift schedule and receive intense treatment^15^, while more prevention-oriented counselling is less resource intensive for both employers and care providers. We thus consider this approach worth exploring, without arguing against the need for more focused treatment options.

## Methods

This prospective, controlled, superiority trial compares outcomes of two parallel groups, namely an online intervention group and a waiting-list control group, randomly assigned in a 1:1 ratio. A third arm was planned to be a face-to-face condition in an outpatient sleep clinic, starting recruitment in 2020 but, due to the Covid pandemic and the associated strain on the health care system, recruitment was halted, and we were forced to adjust the trial and concentrate on the remaining two arms.

### Sample

As discussed in our study protocol^11^, power analyses with the effect size (*d* = 0.76) and correlation among the repeated measures (*r* = 0.67) from our feasibility study^10^ resulted in a required total sample size of *N* = 15 using the ANOVA repeated measures, within-between interaction setting of the G*Power software^16^ for a three-arm design. Adjusting this to two arms and expecting a maximum dropout rate of 50%, as observed in the feasibility study, we aimed to allocate 40 and evaluate at least 20 participants.

We worked with six German industry partners to recruit our sample: Two hospitals, one group of elderly care facilities, and three engineering and manufacturing companies, in which shift work was practiced for a relevant part of the workforce and with which contact had been established in previous collaborations. Company physicians and social counselling services in these companies received screening instructions and lists of access codes to our online platform, which they forwarded to those interested in participating in the trial and meeting eligibility criteria (see Table 1). Informed consent was also obtained at this point. Recruitment was carried out from January 2020 until June 2022. By December 2021, we decided to open recruitment to the public by setting up a website on which anyone could apply to participate in the study. Those interested were screened via e-mail by the counsellors and provided informed consent before receiving an access code and being able to register with the counselling website.

**Table 1:** Eligibility criteria for participants.

Inclusion criteria
Age ≥ 18
Regularly working outside the hours of 6 a.m. to 10 p.m. (> 6 times per month)
Subjective sleep issues revealed in the screening process
Exclusion criteria
Acute suicidality as measured by the respective item in the BDI-II at T0
Ongoing alcohol or drug abuse
No access to required technology (PC, smartphone, tablet)

*Subjective sleep issues*: We constructed a simple nine item symptom checklist to screen for subjective sleep issues. Sample questions are “Do you think about your sleep problems often?”, “Does it take you longer than 30 minutes to fall asleep?”, and “Are you tired throughout the day?”. Possible responses were “Yes” or “No”. Participation was recommended if six or more symptoms were present. *Ongoing alcohol or drug abuse*: Alcohol use was screened for using the respective question in the weekly sleep diaries. More than seven drinks in one week or less than three days without any alcohol consumption in one week would have resulted in exclusion. This did not occur in our sample. Drug use was screened for using a single self-report question: “Do you use any substances to help you sleep?”. Illicit drug use or misuse of prescription medication would have resulted in exclusion. None of the potential participants reported any drug use beyond prescription medication used as prescribed. *BDI II*: Beck Depression Inventory.

After being screened and having received an access code, potential participants were able to anonymously register a profile on our counselling platform. Only an email address was necessary, and we specifically instructed participants to provide an address that did not contain names or other personal information. Upon registration, participants first completed the psychometric assessment at T0 via an online questionnaire on the platform. Their T0 responses were assigned to a trained psychologist who evaluated them and, provided they met all criteria, initiated the counselling process with a personalized message.

Randomization was handled by an employee of the Institute for Online Counselling who was otherwise not associated with the project by randomly assigning all access codes to either the intervention or the waiting list control group in a 1:1 ratio by using Microsoft Excel 2010’s RAND function, a random number generator which uses a Mersenne Twister algorithm^17^. This was done before the codes were given out to the company contact persons, who had no knowledge of this assignment. The assigned counselor had to have knowledge of the assignment to provide the corresponding treatment.

**Fig. 1:**
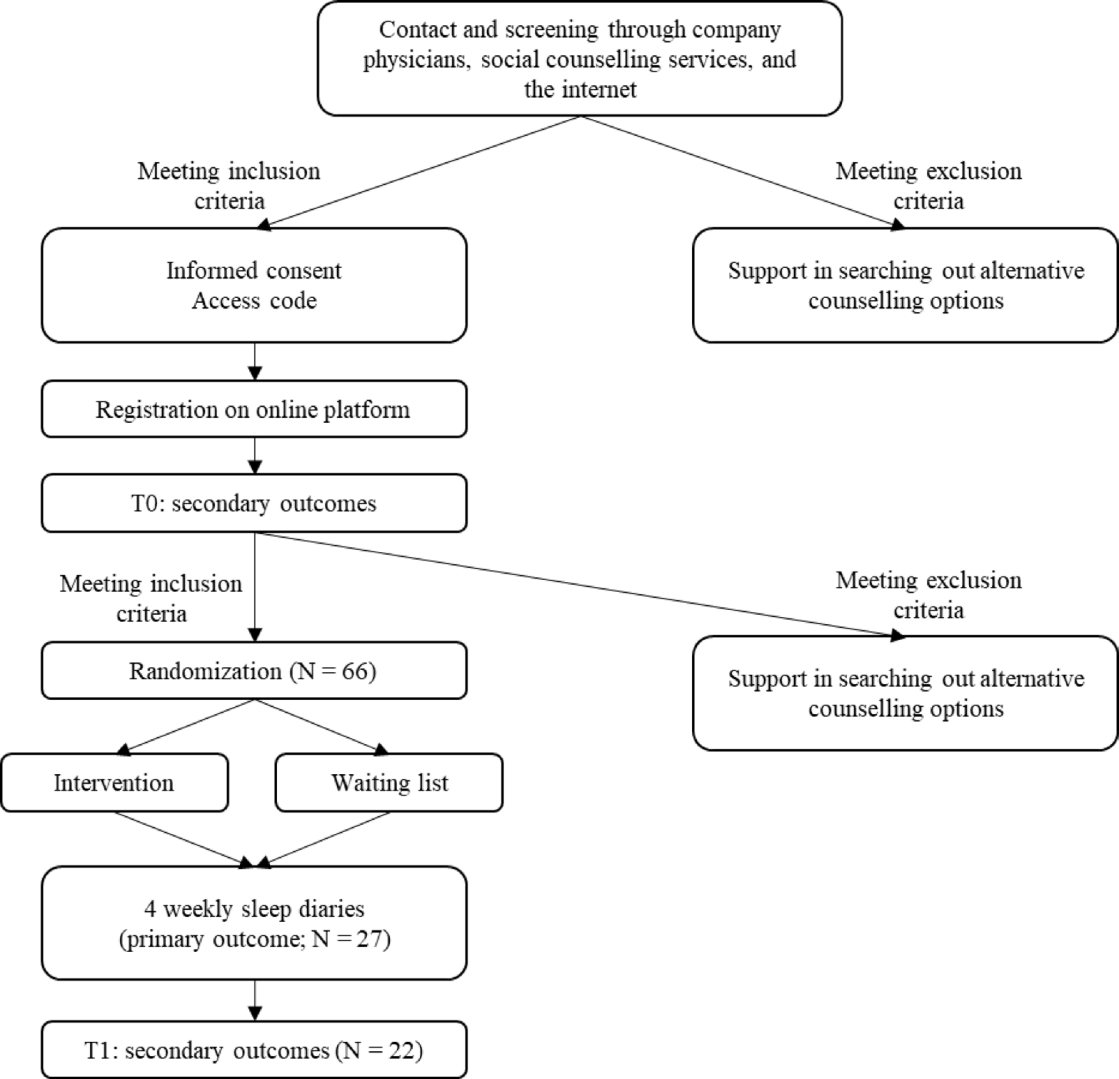
Recruitment flowchart.

A total of 66 shift workers registered an account with an access code on our counselling website and completed T0 measurements, with 44 being recruited by our industry partners (manufacturing: *n* = 35; hospitals and eldercare: *n* = 9) and 22 participating from the public. Forty participants completed the first sleep diary, 33 also completed the second, 29 completed the third, and 27 completed at least 4 sleep diaries (treatment group: *n* = 15; waiting list control group: *n* = 12), providing complete data for our primary outcome and thus representing the per protocol sample for this study (see Fig. 1). We report an average attrition rate of 12.17% per week and a total dropout rate of 59.09%, which may seem high but is generally in the magnitude of other online counselling studies with shift workers^10^ or in the general population^18,19^ and still allowed us to exceed the required sample size per our power analyses. Gender distribution was relatively even (59% male, 41% female) and average age was 43.78 years (*SD* = 12.64 years; range: 23–60 years). Average length of counseling in the treatment group was 36.6 days (*SD* = 11.86 days; range: 27–69 days), which corresponds to our four-week treatment plan and coincides well with the length of the waiting period in the control group (*M* = 36.33 days; *SD* = 9.64 days).

Another five participants dropped out of the study before T1 measurements for the secondary outcomes. This results in 22 participants with complete data sets for T0, at least four sleep diaries, and T1 (treatment group: *n* = 12; waiting list control group: *n* = 10).

### Intervention

**Fig. 2:**
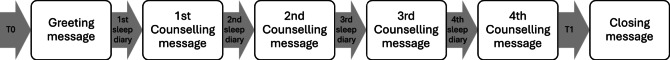
Counselling process and measurement times.

Figure 2 illustrates the study design and the counselling process. After T0, participants in both groups received personalized feedback on their reported symptoms and further information on the next steps, including the result of their randomization, as well as an introduction to the topic of sleep with some basic material about sleep phases, sleep cycles, and sleep need. Both groups were instructed to start their first of four weeks of sleep diaries through our platform. The intervention group was additionally asked to provide some more background about their sleep issues in text format, so as to initiate a conversation with the counsellor.

When the first week of sleep diaries was completed, participants in the intervention group who reported an average sleep efficiency below 85% received personalized instructions for sleep restriction^20^, which they were told to start immediately. The main principles of sleep restriction were followed, including limiting time in bed to last week’s average total sleep time, as well as refraining from naps outside of the agreed upon bedtime. However, in accordance with our study protocol^11^, we did not recommend strictly regular bedtimes, as these would have been impossible to maintain with shift changes. Instead, we individually adapted participants’ sleep windows to their shift schedule, focusing on anchor sleep, i.e. the longest possible overlap between periods spent in bed. We also allowed for splitting sleep windows into two intervals per 24 h cycle, especially before and after night shifts. Directly before and after shift changes or when significant sleep debt (> 1 h per 24 h period) had occurred we allowed for some additional flexibility regarding the timing (± up to 3 h) and length of participants’ bedtimes (± up to 1.5 h). These changes to the classic sleep restriction protocol made it possible to adapt the method to practically any shift schedule we encountered. Participants in the intervention group who reported an average sleep efficiency above 85% were instructed to adhere to regular bedtimes as much as possible, without restricting their time in bed. If at any point in the process their average sleep efficiency dropped below 85%, they were then instructed to start sleep restriction.

After the second week of sleep diaries, participants in the intervention group received personalized sleep hygiene tips, tailored to their specific issues. Depending on the information provided by the participants in their first two sleep diaries and messages up to this point, this could include information on alcohol and caffeine consumption, exercise, nighttime routines, and other helpful habits, as well as optimal bedroom conditions. Counsellors specifically made it a point not to provide generic material and instead acknowledge individual circumstances and behaviors.

After the third week, relaxation techniques were introduced to all participants in the intervention group. We provided a downloadable sound recording for Progressive Muscle Relaxation and an illustration for a simple breathing technique but also instructed participants to seek out other forms of relaxation on the internet, if they had any issue with those two.

After the fourth week of sleep diaries, participants in the intervention group received personalized feedback on their progress up to this point and instructions on how to proceed with the contents that had been introduced.

Every weekly message in the intervention group also contained personalized feedback on the previous week’s sleep diary, including calculations of the average sleep time and time in bed, as well as possible strategies regarding regularity, reported substance use habits, naps or other details. If sleep restriction had been introduced, the messages also included instructions on how to proceed, reducing participants’ time in bed further by 15 min for the next week if the average sleep efficiency was still below 85%, or adding 15 min if sleep efficiency values were now above 85%^20^. Additionally, every message was used to engage in the ongoing conversation, answering questions about sleep and related health issues, as well as pointing to other resources for topics beyond the scope of the counseling process.

Participants in the waiting list control group also received a message after each of their first four weekly sleep diaries, thanking them for their ongoing participation and motivating them to stay in the trial.

After four weeks of sleep diaries, participants in both groups completed the psychometric assessment at T1, repeating all measures from T0. The waiting list control group was then invited to start their counselling process, equivalent to the one described above for the intervention group. All counselling processes were allowed to continue after four weeks, if the participant wished to remain in contact with their counsellor. The two trained counsellors both reported an average total workload of two hours per participant in the intervention group.

### Measures

The primary outcome of this study is the average weekly sleep efficiency as measured by digital sleep diaries. Participants reported the time they went to bed, the time they got up, and their subjective total sleep time for every major sleep period. Additional naps were also reported. Average weekly sleep efficiency was calculated by averaging time in bed (TIB) and total sleep time (TST) over seven consecutive reported sleep periods and dividing TST by TIB. This results in a number between 0 and 1, which is expressed as a percentage between 0% and 100%.

Sleep diaries were also used to monitor for the only potential adverse events we expected, which was defined as a critical drop in TST below 4 h/night across one week^11^. Had such an event occurred, the counselling process would have been adjusted.

The secondary outcomes are the following psychometric measurements collected before (T0) and after (T1) four weeks of sleep diaries.

*Symptoms of depression*. Symptoms of depression were measured using the Beck Depression Inventory II (BDI-II)^21^. The BDI-II consists of 21 items, each employing four statements about a specific symptom, sorted on a scale from 0 to 3, with higher scores corresponding to more severe symptoms. The resulting range of possible scores is 0–63, where a score above 13 indicates mild depression and a score above 19 indicates moderate depression. A sample item is: “Sadness. (1) I do not feel sad. (2) I feel sad much of the time. (3) I am sad all the time. (4) I am so sad or unhappy that I can’t stand it.”.

*Symptoms of insomnia*. We measured symptoms of insomnia with the Regensburg Insomnia Scale (RIS)^22^, a 10-item rating scale to assess the cognitive, behavioral, and emotional aspects of psychophysiological insomnia. Responses are rated on a scale of “0—never” to “4—always”, resulting in a range of possible scores between 0 and 40, where higher scores indicate more severe symptoms of insomnia. Scores above 12 indicate clinically relevant symptoms of insomnia. A sample item is “My sleep is disturbed.”

*Daytime sleepiness.* Daytime sleepiness was measured with the Epworth Sleepiness Scale (ESS)^23^. The test consists of eight items, each describing a situation and asking about the usual chances of dozing off on a scale of “0—would never doze” to “3—high chance of dozing”. The range of possible scores is thus 0–24, with higher scores indicating higher degrees of sleepiness. Scores above 10 are indicative of clinically relevant sleepiness and possible sleep disordered breathing. A sample item is “Sitting quietly after a lunch without alcohol.”

### Analysis

As per our study protocol, we report Intention to Treat (ITT) analyses. To enable these, we applied missing values imputation^24^. First, we checked to see whether *missing completely at random* (MCAR) conditions were met^25^, which was confirmed by Little’s test (χ^2^(35) = 33.62, *p* = 0.54). Then we employed the Expectation-Maximization algorithm from the IBM SPSS Statistics software to impute all missing data regarding our study variables, resulting in a sample size of *n* = 66. To test for group differences in changes in primary and secondary outcomes over time, we employed a repeated measures multivariate analysis of variance (rm-MANOVA) in IBM SPSS Statistics (Version 29).

## Results

Baseline characteristics of our sample at T0 are shown in Table 2. There were no significant group differences in gender distribution, age or T0 measurements.

**Table 2:** Baseline characteristics of the study participants.

Variable	Intervention group (*n* = 37)		Control group (*n* = 29)		Total (*n* = 66)		Tests of group differences		
							χ^2^	t	*p*
Age, *M* (*SD*)	42.27	(11.81)	47.14	(10.93)	44.41	(11.60)		1.72	0.12
Sex							2.50		0.11
Female, *n* (%)	12	(32.4)	15	(51.7)	27	(40.9)			
Male, *n* (%)	25	(67.6)	14	(48.3)	39	(59.1)			
Sleep efficiency, *M* (*SD*)	82.2%	(13.3%)	78.9%	(15.6%)	80.7%	(14.3%)		−0.93	0.35
BDI II sum score, *M* (*SD*)	17.00	(9.84)	17.97	(10.61)	17.42	(10.22)		0.38	0.71
RIS sum score, *M* (*SD*)	19.11	(5.21)	18.93	(5.30)	19.03	(5.21)		−0.14	0.89
ESS sum score, *M* (*SD*)	9.38	(4.86)	10.41	(4.75)	9.83	(4.81)		0.87	0.39

BDI II, Beck depression inventory^21^; RIS Regensburg insomnia scale^22^; ESS Epworth sleepiness scale^23^.

The results of the rm-MANOVA are displayed in Table 3. We identified significant changes over time regarding sleep efficiency (*F*(1) = 40.73, *p* < 0.001), symptoms of depression (*F*(1) = 100.37, *p* < 0.001), and symptoms of insomnia (*F*(1) = 60.47, *p* = 0.001), but not daytime sleepiness (*F*(1) = 0.40, *p* = 0.53). These changes, however, were independent of participants’ treatment group, as none of the time x group interactions reached statistical significance (all *p* > 0.10).

**Table 3:** Means, standard deviations, and rm-MANOVA statistics for primary and secondary outcomes in the ITT sample.

Variable	Intervention group (*n* = 37)		Control group (*n* = 29)		ANOVA			
					Effect	F	df	*p*
Sleep efficiency, *M* (*SD*)								
t0	82.2%	(13.3%)	78.9%	(15.6%)	G	0.76	1	0.39
t1	85.7%	(9.6%)	83.7%	(11.9%)	T	40.73	1	**< 0.001**
					G x T	0.96	1	0.33
BDI II sum score, *M* (*SD*)								
t0	17.00	(10.61)	17.97	(9.84)	G	0.38	1	0.54
t1	8.72	(6.70)	10.20	(6.21)	T	100.37	1	**< 0.001**
					G x T	0.10	1	0.75
RIS sum score, *M* (*SD*)								
t0	19.11	(5.21)	18.93	(5.30)	G	0.01	1	0.93
t1	14.24	(5.55)	14.18	(6.16)	T	60.47	1	**< 0.001**
					G x T	0.01	1	0.92
ESS sum score, *M* (*SD*)								
t0	9.38	(4.86)	10.41	(4.75)	G	1.01	1	0.32
t1	9.22	(3.09)	10.09	(3.58)	T	0.40	1	0.53
					G x T	0.05	1	0.83

BDI II, Beck depression inventory [21]; RIS: Regensburg insomnia scale [22]. ESS: Epworth sleepiness scale [23]. *p*-values < 0.05 are printed in bold.

Regarding our primary outcome, post-hoc analyses showed significant increases in sleep efficiency in the treatment group by an average of 3.6% (*t*(36) = −4.02, *p* < 0.001), as well as the control group by an average of 4.9% (*t*(28) = −5.00, *p* < 0.001). There were no significant associations between improvements in sleep efficiency and any measures collected at T0, including participant age or gender (all *p* > 0.10). Sleep efficiency improvements were also statistically independent of the length of the counselling (treatment group) or the length of the waiting period (control group).

For our secondary outcomes, we identified significant improvements regarding symptoms of insomnia (*t*(36) = 6.45, *p* < 0.001) and depression (*t*(36) = 7.75, *p* < 0.001) in the treatment group, as well as in the control group (insomnia: *t*(28) = 4.83, *p* < 0.001; depression: *t*(28) = 6.53, *p* < 0.001).

We calculated Cohen’s d effect sizes for all significant improvements. In the treatment group, this resulted in a medium effect size for sleep efficiency (*d* = 0.66) and large effect sizes for symptoms of depression (*d* = 1.28) and insomnia (*d* = 1.03). The improvements in the control group were all equivalent to large effect sizes (sleep efficiency: *d* = 0.93; insomnia: *d* = 0.90; depression: *d* = 1.21).

No adverse events occurred during the trial which would have necessitated deviations from the study protocol.

### Exploratory analyses

We additionally report results for the per-protocol sample of 27 participants without imputation. The results are displayed in Table 4. Again, analyses of variance identified significant changes over time regarding sleep efficiency (*F*(1) = 15.77, *p* < 0.001), symptoms of depression (*F*(1) = 17.23, *p* < 0.001), and symptoms of insomnia (*F*(1) = 7.40, *p* = 0.013), but not daytime sleepiness (*F*(1) = 0.13, *p* = 0.726). Here, too, none of the time x group interactions reached statistical significance (all *p* > 0.10).

Next, we checked to see whether these null results were caused by a ceiling effect due to the twelve subclinical cases in our per-protocol sample of 27 participants [26]. We repeated the ANOVA analyses after excluding all cases who reported sleep efficiencies above the clinical threshold of 85% in the first week of sleep diaries, leaving us with a sample size of *n* = 15 (treatment group: *n* = 6; waiting list control group: *n* = 9). Results remained unchanged, both in the original sample and when the same exclusion criterion was applied to the imputed sample, with significant time effects for all outcomes except daytime sleepiness, but no significant time x group interactions.

Lastly, we report the results of two additional subjective measures in the treatment group, which we measured using one item each at T1: participant satisfaction (“I am satisfied with the counselling”) and perceived helpfulness (“The counselling helped me a lot.”). Responses were recorded on a scale of “1—not at all” to “4—completely”. Average satisfaction in the treatment group was 3.58 (*SD* = 0.52), with no participants choosing less than a 3 on our 4-point scale. Average perceived helpfulness was 2.92 (*SD* = 0.90), with 75% of participants choosing a 3 or a 4 on the scale.

**Table 4:** Means, standard deviations, rm-ANOVA statistics for primary, and rm-MANOVA statistics for secondary outcomes in the ITT sample.

Variable	Intervention group (*n* = 15)		Control group (*n* = 12)		ANOVA			
					Effect	F	df	*p*
Sleep efficiency, *M* (*SD*)								
t0	83.5%	(12.8%)	76.0%	(14.2%)	G	2.01	1	0.17
t1	86.7%	(10.1%)	81.0%	(12.3%)	T	15.77	1	**< 0.001**
					G × T	0.73	1	0.40

	Intervention group (*n* = 12)		Control group (*n* = 10)					
BDI II sum score, *M* (*SD*)								
t0	15.42	(9.40)	12.70	(7.62)	G	0.06	1	0.81
t1	7.42	(7.80)	8.60	(6.60)	T	17.23	1	**< 0.001**
					G × T	1.79	1	0.20
RIS sum score, *M* (*SD*)								
t0	17.75	(3.77)	18.60	(4.67)	G	1.69	1	0.21
t1	13.25	(5.89)	17.10	(5.20)	T	7.39	1	**0.01**
					G × T	1.85	1	0.19
ESS sum score, *M* (*SD*)								
t0	7.58	(4.58)	9.60	(4.03)	G	1.91	1	0.18
t1	7.17	(2.66)	9.50	(4.72)	T	0.13	1	0.73
					G × T	0.05	1	0.83

BDI II, Beck depression inventory^21^; RIS, Regensburg insomnia scale^22^; ESS, Epworth sleepiness scale^23^. *p*-values < 0.05 are printed in bold.

## Discussion

This study explored the effects of a four-week online sleep counselling program for shift workers on sleep efficiency, symptoms of depression and insomnia, as well as daytime sleepiness in a randomized controlled trial. Results show improvements regarding sleep efficiency, as well as symptoms of depression and insomnia, but, unexpectedly, no group effect on outcome changes between measurements. Neither repeating the analyses using only the per-protocol sample, nor excluding sub-clinical cases changed this result. Post hoc analyses in the ITT study sample show significant improvements in sleep efficiency, symptoms of insomnia and depression, but not daytime sleepiness, in the treatment group as well as the control group.

Since none of the time x group interactions reached significance, we do not interpret these results as group differences. When looking for an explanation for the improvements across both groups, we first look at what participants in both groups had in common: Both groups were instructed to report information about daily sleep behaviors in sleep diaries for four weeks. Research has shown that self-monitoring can be advantageous even without professional assistance^27^. Through periodic measurements, awareness of relevant symptoms, sensations, and behaviors is increased, enhancing positive health behaviors. Specifically, the increase in sleep efficiency in the control group might be explained by improved self-management, as participants focused more attention on their sleep behaviors, independently noticed suboptimal routines, and consequently adjusted the times they went to bed and got up. Future research should address this finding by testing low threshold self-guided sleep tracking interventions for shift workers, which might be a viable option for primary and secondary prevention of sleep disorders. Specifically, sleep diaries, which we argue require deeper attention to one’s own sleep behavior, could be compared to the use of wearable sleep trackers. The finding also shows the importance of employing an active control group in sleep intervention research, so as not to overestimate the effectiveness of a treatment.

The lack of improvement regarding daytime sleepiness, while not expected, sits well with other findings in the field^28^, including our own pilot study^10^. Possible explanations for this result could be that daytime sleepiness is not a core symptom of insomnia in the first place^28^ and that transient increases in sleepiness can be side effects of some of the tools of Cognitive Behavioral Therapy for Insomnia^14,19^.

The main limitation of our study lies in the time frame. Our design lacks a follow-up measurement which would have enabled us to test long-term developments in sleep quality, but which also would have necessitated withholding the intervention in our waiting list control group for months, which we deemed unethical. The tools and contents of Cognitive Behavioral Therapy for Insomnia, which our intervention is rooted in, are designed to help patients prevent relapses in the months and years following treatment success^29^. We assume our program will have the same positive long-term effect, but we have no way of proving this.

However, we would not necessarily expect a longer time frame to increase group differences, as the positive effects of cognitive behavioral approaches to sleep improvement typically decline over time^30^. In searching for other adjustments to our intervention that could lead to improvements above and beyond those in an active control group, we identified three options for future research. Firstly, sacrificing a degree of anonymity by augmenting the text based, asynchronous online intervention with some form of more immediate counsellor contact, such as phone or video calls could further improve participants’ motivation and adherence, thereby in turn increasing effectiveness^31^. Secondly, specifically for symptoms of depression, adding a social element to the intervention, such as an online discussion board for exchanging ideas and connecting with others, inside and outside the organization, could bring another helpful dimension to the program [5], which could offset some of the negative effects of the social jetlag associated with shift work and facilitate the flow of knowledge about helpful coping strategies. Thirdly, increasing statistical power by recruiting larger samples could lead to more robust effects, as shown by studies of online sleep interventions for non-shift workers with similar effect sizes, but larger samples^32^. While we exceeded our goal of 20 participants, the effect size for our primary outcome in this study (*d* = 0.66) was smaller than the one we observed in our pilot study (*d* = 0.76)^10^, which was the foundation for our power analyses. We also have to acknowledge that Cognitive Behavioral Therapy for Insomnia, while shown to be promising as a framework for improving sleep issues in shift workers^9,10^, is not originally designed for this population. While we attempted to modify its contents to fit shift workers’ needs and individually tailored our intervention to every participant, more extreme modifications or entirely new approaches might be necessary. For example, a stronger focus on relaxation techniques, the introduction of mindfulness practices, or dropping sleep restriction from the intervention might be explored in future research.

Another limitation of this study lies in its dependence on subjective measures. Due to the anonymity we provided our participants, we were unable to employ actigraphy devices or other objective sleep measures. We suggest future research augment self-report measures with more objective data whenever possible.

Unfortunately, recruitment for this study started in January of 2020, only a few weeks before the Covid-19 pandemic hit Germany and most of the world implemented radical lockdown measures. This led to recruitment problems in both industries we mainly worked with. In health- and eldercare, workers found themselves on the frontline of a global crisis^33^, experiencing high rates of stress, anxiety, and depression^34^, and were thus certainly occupied with more pressing issues than their own sleep health. In engineering and manufacturing, working hours were temporarily reduced in many organizations, coinciding with a drop in manufacturing output^35^. This, somewhat paradoxically, might have led to improvements in sleep and general well-being in those who suddenly had to work less shifts, as they were able to make decisions about their sleeping times more freely and got to spend more quality time with loved ones^36^, leading to lower motivation to participate in an intervention such as ours. In addition, our main contact persons in those organizations—company physicians and counselling personnel—reported having other, more pressing issues to attend to than recruiting for our intervention during that time. We continued recruitment well into 2022, when healthcare systems and manufacturing operations had largely rebounded from the peak of the pandemic, but our results still need to be interpreted with the background of a global crisis.

While we acknowledge these limitations, the strengths of this study lie in the core issues addressed in the introduction: Methodologically, by employing a randomized controlled trial design, we’re able to generate stronger causal inference compared to uncontrolled studies. Relying on highly individualized instead of generic or algorithmically derived contents and the guidance of trained and experienced counsellors enabled us to provide a personalized intervention to every participant instead of a one-size-fits-all approach. At the same time, we were able to uphold anonymity and asynchronicity, which are highly valued by shift workers in our experience. Finally, by including subclinical cases, we want to shift the focus of sleep interventions for shift workers towards prevention, which has long been argued for in the literature^5,37,38^.

In summary, we show that a four-week online intervention for sleep problems in shift workers, rooted in the cognitive behavioral approach, but focusing on prevention and personalization, is feasible. However, we cannot demonstrate effectiveness above and beyond a waiting list control group. While we show moderate to large effect sizes in improving sleep efficiency as well as symptoms of depression and insomnia, we cannot directly attribute these to our intervention. The improvements we observed might have been caused by repeated sleep diary completion, which should be explored as a low threshold intervention for shift workers.

## Data Availability

Because the sample is small and we guaranteed anonymity to our participants, we cannot provide our study data publicly. Upon reasonable request, we will provide a reduced dataset containing no demographic data.
